# Targeted RNA-sequencing analysis for fusion transcripts detection in tumor diagnostics: assessment of bioinformatic tools reliability in FFPE samples

**DOI:** 10.37349/etat.2022.00102

**Published:** 2022-10-27

**Authors:** Iolanda Capone, Fabio Bozzi, Gian Paolo Dagrada, Paolo Verderio, Elena Conca, Adele Busico, Maria Adele Testi, Valentina Monti, Matteo Duca, Claudia Proto, Silvia Damian, Alberta Piccolo, Federica Perrone, Elena Tamborini, Andrea Devecchi, Paola Collini, Daniele Lorenzini, Andrea Vingiani, Luca Agnelli, Giancarlo Pruneri

**Affiliations:** 1 Department of Pathology and Laboratory Medicine, Fondazione IRCCS Istituto Nazionale dei Tumori, 20133 Milan, Italy; 2 Department of Applied Research and Technological Development, Fondazione IRCCS Istituto Nazionale dei Tumori, 20133 Milan, Italy; 3 Department of Medical Oncology, Fondazione IRCCS Istituto Nazionale dei Tumori, 20133 Milan, Italy; 4 Department of Oncology and Hemato-oncology, University of Milan, 20133 Milan, Italy; Université Côte d’Azur; Nice University Hospital, France

**Keywords:** Next-generation sequencing (NGS), RNA-sequencing, fluorescence *in situ* hybridization (FISH), formalin-fixed paraffin-embedded (FFPE), gene fusions

## Abstract

**Aim:**

Diagnostic laboratories are progressively introducing next-generation sequencing (NGS) technologies in the routine workflow to meet the increasing clinical need for comprehensive molecular characterization in cancer patients for diagnosis and precision medicine, including fusion-transcripts detection. Nevertheless, the low quality of messenger RNA (mRNA) extracted from formalin-fixed paraffin-embedded (FFPE) samples may affect the transition from traditional single-gene testing approaches [like fluorescence *in situ* hybridization (FISH), immunohistochemistry (IHC), or polymerase chain reaction (PCR)] to NGS. The present study is aimed at assessing the overall accuracy of RNA fusion transcripts detection by NGS analysis in FFPE samples in real-world diagnostics.

**Methods:**

Herein, NGS data from 190 soft tissue tumors (STTs) and carcinoma cases, discussed in the context of the institutional Molecular Tumor Board, are reported and analyzed by FusionPlex^©^ Solid tumor kit through the manufacturer’s pipeline and by two well-known fast and accurate open-source tools [Arriba (ARR) and spliced transcripts alignment to reference (STAR)-fusion (SFU)].

**Results:**

The combination of FusionPlex^©^ Solid tumor with ArcherDX^®^ Analysis suite (ADx) analysis package has been proven to be sensitive and specific in STT samples, while partial loss of sensitivity has been found in carcinoma specimens.

**Conclusions:**

Albeit ARR and SFU showed lower sensitivity, the use of additional fusion-detection tools can contribute to reinforcing or extending the output obtained by ADx, particularly in the case of low-quality input data. Overall, our results sustain the clinical use of NGS for the detection of fusion transcripts in FFPE material.

## Introduction

Historically, recurring histotype-specific translocations have been reported in hematological malignancies and soft tissue tumors (STTs) and a growing number of gene fusions have been identified also in different types of carcinomas [1]. Such fusions not only drive tumorigenesis but also represent a powerful tool for diagnosis and potential targets for personalized therapy, with several drugs now constituting standard-of-care across malignancies. Fluorescence *in situ* hybridization (FISH) and reverse-transcription polymerase chain reaction (RT-PCR) are currently recognized as the standard methods for detecting gene fusions in formalin-fixed paraffin-embedded (FFPE) specimens, since they are affordable and widely distributed. Nevertheless, these technologies have a number of limitations, above all if the partner gene is unknown: since they test either single gene break apart (FISH) or specific fusions (FISH and RT-PCR), whenever several fusions of interest are present the analysis would result in time- and cost-consuming. Immunohistochemistry (IHC) has been recently introduced as a surrogate diagnostic test, being able to reliably detect fusion events due to chimeric protein overexpression [2–4], but has been approved for clinical use only in peculiar settings [5].

Although FISH, IHC and/or RT-PCR are still the most frequently used diagnostic adjunct (i.e. EWS RNA binding protein 1 (*EWSR1*)-friend leukemia virus integration 1 (*FLI1*) fusion in Ewing sarcoma), the introduction of next-generation sequencing (NGS) technologies has dramatically improved the scenario, particularly in cases requiring multiple testing, where NGS is cost-effective in identifying known and novel fusions genes [6, 7]. NGS analysis requires peculiar skills and dedicated platforms, which are currently limited to reference labs albeit available at ever-lower costs.

On the other hand, the “Achille’s heel” of NGS workflow for diagnostic purposes is represented by issues related to the quality of DNA/RNA and by computational analysis. Several pre-analytical variables can in fact negatively affect the library quality, including the handling of the surgical specimens and RNA fragmentation related to formalin-fixation (as it happens, moreover, for RT-PCR). Both issues negatively impact nucleic acids quality, introducing unpredictable biases that ultimately lead to low-coverage and/or low-quality reads [8]. Beyond nucleic acids quality, bioinformatic analysis of fusion transcripts is also challenging since all existing methods suffer, at least in part, from poor prediction accuracy. To improve the predictive power, it has been suggested that the results from at least two algorithms should be evaluated [9]. However, combining more approaches also presents disadvantages, since it is computationally expensive and, while improving sensitivity, can affect specificity.

Herein, we report the results obtained in a consecutive series of FFPE tumor specimens analyzed by NGS RNA-sequencing (RNAseq) for the identification of fusion transcripts relevant for diagnosis and/or treatments. The RNA was processed using a commercial assay, the FusionPlex^©^ solid tumor kit (for sarcomas or for carcinomas), and analyzed using the manufacturer’s software package ArcherDx^©^ Analysis suite (ADx). In order to assess the efficacy of integrating multiple fusion-detecting algorithms in a real-world dataset, we used two alternative tools to ADx, i.e. Arriba (ARR) [9] and spliced transcripts alignment to reference (STAR)- fusion (SFU) [10], which have been both recently recognized as highly accurate in fusions detection [11].

## Materials and methods

### Patients samples

The case cohort included 193 consecutive FFPE surgical, bioptic or cytological tumor samples from 190 patients evaluated by RNAseq assays for the detection of gene rearrangements. Sixty-seven percent of the study population were in-patients while the remaining 33% were external patients. In particular: 104 (53.9%) core needle biopsies (CNBs), 84 (43.5%) surgical samples and 5 (2.6%) cytology specimens were analyzed. Informed consent was obtained at the time of admittance in the context of the Institutional Molecular Tumor Board, i.e. the multidisciplinary team that collects, analyzes, discusses, and stores molecular data of patients, mainly with metastatic or locally advanced tumors, who might be addressed to personalized treatment. This study was performed according to the Declaration of Helsinki and approved by the Ethics committee of Fondazione IRCCS Istituto Nazionale Tumori Milano (INT 277/20). FISH data concerning routine diagnostic procedures whenever available were retrieved from the institutional database of the department of pathology.

### IHC

IHC was carried out on FFPE 2 μm sections, using an automated immune-stainer (BenchMark Ultra, Ventana Medical Systems, Inc, Tucson, AZ, USA). ALK-DF53 [Ventana, catalog (cat): 790-4794], tropomyosin receptor kinase (TRK)-pan (Ventana, cat: 790-4795), and their negative control (Ventana, cat: 790-4795) were diluted, incubated, and developed according to manufacturer instructions. ROS-1 (Cell Signaling, cat: 32/87S) was retrieved using 1× EDTA buffer (cat: CB917M, BIOCARE medical, Bioptica Milan Italy) pH 8 in autoclave at 120°C for 10 min, diluted 1:100, incubated at room temperature for 120 min and developed with diaminobenzidine.

### FISH analysis

Both commercial and in-house made [bacterial artificial chromosome (BAC)] probes were employed for FISH experiments (Table S1). The BAC probes were obtained from Children’s Hospital Oakland Research Institute [C.H.O.R.I.; BAC-P1 derived artificial chromosome (PAC) resources, C.H.O.R.I., California], labeled with either Spectrum Green or Spectrum Orange fluorochromes (Abbott Molecular, Des Plains, Illinois) by means of nick translation (Nick Translation Reagent Kit, Abbott Molecular) following manufacturer’s instructions, and validated on normal metaphase from peripheral blood and on FFPE positive controls. The FFPE samples were treated for FISH following standard procedure. A minimum of 50 nuclei were analyzed using a Leica DM 6000B (Wetzlar, Germany) microscope at 100× magnification and the appropriate fluorescence filters. The images were captured using Cytovision software (version 7.0 Leica). The positivity thresholds used were 15% and 10% for break apart and fusion respectively.

### RNA processing, libraries construction and sequencing

After histological quality check and, when necessary, tumor enrichment by micro-dissection, up to ten 2–5 μm sections were cut from representative FFPE specimens. The number of the slices varied according to the size and tumor cellularity and was aimed at reaching the minimum RNA amount request (i.e. 20 ng). The slices were re-hydrated with xylene and alcohols and total nucleic acid was extracted using the Maxwell^®^ RSC RNA FFPE Kit (cat: AS1440, Promega, Milan, Italy) in accordance with the manufacturer’s recommendations. RNA concentration was quantified using Qubit™ RNA High-Sensitive Assay kit on the Qubit™ fluorometer (cat: Q10210, ThermoFisher Scientific, Waltham, MA, USA). For the detection of known and novel fusions, we adopted RNA-based tests, namely the FusionPlex^©^ Sarcoma panel (cat: AB004, Invitae, Boulder, CO, USA) and the FusionPlex^©^ Lung panel (cat: DB0222, Invitae). The sarcoma panel includes the following genes: ALK receptor tyrosine kinase (*ALK*), calmodulin binding transcription activator 1 (*CAMTA1*), cyclin B3 (*CCNB3*), capicua transcriptional repressor (*CIC*), enhancer of polycomb homolog 1 (*EPC1*), *EWSR1*, forkhead box O1 (*FOXO1*), FUS RNA binding protein (*FUS*), GLI family zinc finger 1 (*GLI1*), high mobility group AT-hook 2 (*HMGA2*), JAZF zinc finger 1 (*JAZF1*), MYST/Esa1 associated factor 6 (*MEAF6*), myocardin like 2 (*MKL2*), nuclear receptor coactivator 2 (*NCOA2*), neurotrophic receptor tyrosine kinase 3 (*NTRK3*), platelet derived growth factor subunit B (*PDGFB*), PLAG1 zinc finger (*PLAG1*), ROS proto-oncogene 1 (*ROS1*), SS18 subunit of BAF chromatin remodeling complex (*SS18*), signal transducer and activator of transcription 6 (*STAT6*), TATA-box binding protein associated factor 15 (*TAF15*), transcription factor 12 (*TCF12*), transcription factor binding to IGHM enhancer 3 (*TFE3*), trafficking from ER to golgi regulator (*TFG*), ubiquitin specific peptidase 6 (*USP6*), tyrosine 3-monooxygenase/tryptophan 5-monooxygenase activation protein epsilon (*YWHAE*). The lung panel includes the following genes: *ALK*, B-Raf proto-oncogene (*BRAF*), epidermal growth factor receptor (*EGFR*), fibroblast growth factor receptor 1 (*FGFR1*), *FGFR2*, *FGFR3*, KRAS proto-oncogene (*KRAS*), MET proto-oncogene, receptor tyrosine kinase (*MET*), neuregulin 1 (*NRG1*), *NTRK1*, *NTRK2*, *NTRK3*, ret proto-oncogene (*RET*), and *ROS1*. Depending on the type of sample (CNB, surgical or cytologic samples), the RNA used for complementary DNA (cDNA) synthesis ranged from about 20 ng to 250 ng. This high variability was due to the cohort of samples that comprised CNB with low cellularity. The libraries were quantified using the Qubit™ RNA HR Assay Kit (ThermoFisher Scientific), diluted to 50pM, pooled and loaded on the Ion Chef system (ThermoFisher Scientific) to perform automated template preparation and on-chip loading. The libraries were sequenced with the GeneStudio™ S5 sequencer (Ion Torrent platform, ThermoFisher Scientific). Positive and negative controls are not required by the FusionPlex^©^ manufacturers’ protocol, unless those normally included during library preparation to exclude RNA contamination (no template). The FusionPlex^©^ panel was further validated with a parallel analysis with FISH on FFPE material in sarcomas [12].

### Data analysis

ADx version 6.2.3 was used to analyze the results of the FusionPlex^©^ panels using default settings. Predefined parameters (“QC PASS”) were used to assess the quality of the data, which, according to the ADx user manual, allow up to 95 percent sensitivity in the detection of fusions. Samples that did not pass the quality checks were excluded. Fusions were called if detected as “high confidence calls”. The same raw data were also evaluated by ARR [9] and SFU [10] informatic tools. Among the fusion transcripts detected by these two tools, only those with high reliability were considered. Specifically, we retained only transcripts labeled with “high level” of confidence for ARR (after artifacts removal and candidate fusions filtering based on the number and type of unique supporting reads) and “large” as Anchor Support for SFU (the full pipeline to obtain the “*STAR-Fusion.filter*” file is described in Haas et al. [10]). For convenience, the parameters used for ARR are reported in the Supplementary material.

### Statistical methods

To test the threshold for sensitivity in lung panel, a hypergeometric distribution test was applied, considering progressively increasing thresholds among adjacent values of the distribution of the read counts. In other words, a one-tailed Fisher’s exact test has been applied to measure the statistical significance of randomly gathering number of patients presenting concordant FISH/NGS results from the population stratified into two groups, according to any threshold between the adjacent values in the ordered distribution of read counts. The overall accuracy of ARR and SFU was assessed in terms of sensitivity, specificity, positive (PPV) and negative predictive value (NPV) considering ADx as “reference” method. R software version 4.1.2 has been used to perform all the analyses.

## Results

### RNAseq in the diagnostic workflow

RNAseq analysis for the identification of aberrant transcripts was carried out in a series of 193 FFPE samples obtained from 190 patients. Most of the patients [48.4% (91/190)] were lung adenocarcinomas, 23.2% (43/190) STT, 5.8% (11/190) brain tumors, 2.6% (6/190) neuroendocrine cancers, and 2.1% (4/190) thyroid carcinomas; the remaining 18.4% (35/190) were represented by minor fractions of different tumor types, mainly carcinomas. FISH data were available for 71 cases including: (i) cases undergoing FISH analysis upfront for standard diagnostic procedures and for whom NGS data were thereafter required for the identification of the specific fusion partner (e.g., in the case of *EWSR1*-translocated STT); (ii) cases in which multiple FISH tests did not allow to detect any fusion (mainly in STT); or (iii) cases in which FISH have been used to validate RNAseq data. Two different RNAseq assays were applied, the FusionPlex^©^ sarcoma panel or the FusionPlex^©^ lung panel. The first one was applied to 30 STT plus 1 meningioma while the lung panel was applied to the remaining cases. Since the lung panel included *NTRK 1*, *2*, and *3* was also applied in 13 STTs suspected to be *NTRK* rearranged.

### Sarcoma panel

#### ADx results showed high concordance with FISH data

**Figure 1.**
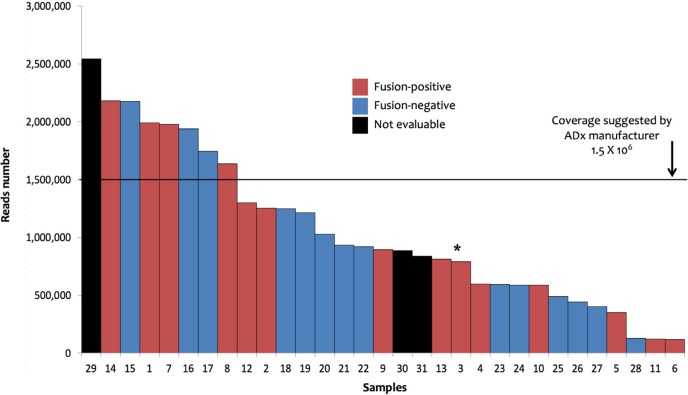
Sarcoma panel: ADx results. The 31 samples investigated with sarcoma panel and analyzed by ADx are shown ordered on the basis of the number of the reads. Sample 3 (marked by the asterisk) showed the *EWSR1-TFCP2* fusion coupled with an ALK large deletion (Δ2-18) as well as ALK unbalanced translocation

Data stemming from the analysis with the FusionPlex^©^ sarcoma panel were analyzed by the ADx software. In this setting, 28/31 processed samples produced 894,000 median reads (range: 116.677–2.542.366), whereas the remaining 3 cases did not pass quality checks. A total of 14 (45%) fusion transcripts plus one ALK large deletion (ALK Δ2-18) were identified in 14 patients (Table 1). The metrics of the analysis of the 31 samples are shown in Figure 1. The fusions detection rate did not depend on reads number [Wilcoxon test, *P*-value no significance (n.s.)]. FISH data were available for 11 of these 14 samples and confirmed 7 fusions, lost 3 chimeric transcripts, and found one *ALK* unbalanced translocation in the case in which ADx showed the large *ALK* deletion (sample 3, Table 1, Figure S1). Among the 4 cases (samples 11, 12, 13, and 14, Table 1) for which FISH results were not available, three (11, 12, and 13) tumors presented known recurrent fusion events: *EWSR1*-cAMP responsive element binding protein 3 like 2 (*CREB3L2*) fusion in sclerosing epithelioid fibrosarcoma [13], *EWSR1-FLI1* in a case of Ewing sarcoma, and NGFI-A binding protein 2 (*NAB2*)-*STAT6* in a solitary fibrous tumor. The ETS variant transcription factor 6 (*ETV6*)-*NTRK3* fusion (sample 14, Table 1) was detected in a cellular mesoblastic nephroma in which screening IHC had pan-TRK nuclear immunoreactivity. The three cases with discordant results (ADx positive/FISH negative, samples 8, 9, and 10, Table 1) likely bore cryptic fusions, i.e. subtle genomic rearrangements, such as insertions, recognized as potential pathogenic events that can be missed by FISH [14–16]. Finally, among the 14 ADx negative and the 3 not evaluable samples analyzed with the sarcoma panel, no fusions were detected by FISH in 12/17 cases (samples 15–31, Table 1). Collectively, these data validate the clinical utility of ADx in STT samples.

**Table 1.** Table showing the gene fusions called by ADx in the 31 cases analyzed by sarcoma panel

**Samples**	Histology/Diagnosis*	**Sarcoma panel ADx**	**FISH**
1	Inflammatory myofibroblastic tumor^a^	*ETV6-NTRK3*	*ETV6*-*NTRK3*
2	Myoepithelial carcinoma	*EWSR1-PATZ1*	*EWSR1* translocated
3	EWSR1-TFCP2 rearranged sarcoma	*EWSR1-TFCP2* *ALK* deletion (Δ2-18)	*EWSR1* translocated *ALK* translocated
4	Extraskeletal myxoid chondrosarcoma	*EWSR1-NR4A*3	*EWSR1-NR4A3*
5	Ewing sarcoma	*EWSR1-FLI1*	*EWSR1-FLI1*
6	EWS-rearranged small round cells sarcoma	*EWSR1-CREM*	*EWSR1* translocated
7	High grade ESS	*YWHAE-NUTM2B*	*YWHAE* translocated
8	Monophasic synovial sarcoma	*SS18-SSX4* ^b^	*SS18* intragenic rearrangement
9	Biphasic synovial sarcoma	*SS18-SSX1* ^b^	*SS18* not translocated
10	CIC-rearranged sarcoma	*CIC-DUX4* ^c^	*CIC* not translocated
11	Sclerosing epithelioid fibrosarcoma	*EWSR1-CREB3L2*	Not done
12	Ewing sarcoma	*EWSR1-FLI1*	Not done
13	Solitary fibrous tumor	*NAB2-STAT6* ^d^	Not detectable by FISH
14	Cellular congenital mesoblastic nephroma	*ETV6-NTRK3*	Not done
15	MPNST	No fusions	*SS18, EWSR1, FUS* negative
16	Composite hemangioendothelioma	No fusions	*WWTR1* negative^e^
17	Myoepithelial carcinoma	No fusions	*SS18, BCOR, CIC* negative
18	Undifferentiated sarcoma, NOS	No fusions	*EWSR1, FUS* negative^e^
19	Embryonal rhabdomyosarcoma	No fusions	*SS18* negative
20	Angiomatoid fibrous histiocytoma	No fusions	*EWSR1, FUS, ATF1, CREB*1 negative
21	Pleomorphic sarcoma	No fusions	Not done
22	Undifferentiated small round cell sarcoma	No fusions	*EWSR1*, *BCOR*, *FUS*, *CIC*, *NCOA2* negative^e^
23	Undifferentiated sarcoma, NOS	No fusions	Not done
24	Undifferentiated sarcoma, NOS	No fusions	Not done
25	Spindle cell sarcoma, NOS	No fusions	*FUS* negative^e^
26	Meningioma	No fusions	Not done
27	Sclerosing epithelioid fibrosarcoma^f^	No fusions	*ALK* negative
28	Malignant myopericytoma	No fusions	*SS18*, *EWSR1*, *BCOR*, *CIC*, *TFE3* negative
29	Myoepithelioma	Not evaluable	*PHF1*, *FUS*, *CIC, BCOR, EWSR1, NR4A3* negative
30	High grade sarcoma, NOS	Not evaluable	*EWSR1*, *NCOA2*, *CIC*, *SS18*, *BCOR* negative
31	Oral cavity neoplasm^g^	Not evaluable	Not done

a: the ETV6-NTRK3 translocation is unusual in inflammatory myofibroblastic tumor and was found in a lung biopsy obtained from a male 67 years hold patient. b: the SS18-SSX fusions were found in samples from 2 patients that had clinical histories and immunophenotypes fitting synovial sarcoma. In one of these two cases (sample 8), FISH revealed a pattern of SS18 intragenic rearrangement suggestive of SS18 translocation. As described in the main text, SS18 cryptic fusions undetectable by FISH, are well-known events. c: the same situation described in the previous note can be drawn for *CIC* gene. d: NAB2- STAT6 is a translocation not detectable by FISH. e: RNAseq was performed on surgical samples while FISH was done on CNBs prior surgery. f: external diagnosis. g: final diagnosis not available. *: According to WHO classification of Soft tissue and bone tumors [17]; ESS: endometrial stromal sarcoma; MPNST: malignant peripheral nerve sheet tumor; NOS: not otherwise specified; *PATZ1*: POZ/BTB and AT hook containing zinc finger 1; *TFCP2*: transcription factor CP2; *NR4A3*: nuclear receptor subfamily 4 group A member 3; *CREM*: cAMP responsive element modulator; *NUTM2B*: NUT family member 2B; *SSX4*: SSX family member 4; *DUX4*: double homeobox 4; *WWTR1*: WW domain containing transcription regulator 1; *BCOR*: BCL6 corepressor; *ATF1*: activating transcription factor 1; *CREB1*: cAMP responsive element binding protein 1; *PHF1*: PHD finger protein 1

#### ARR and SFU showed sensitivity loss in comparison to ADx

**Figure 2.**
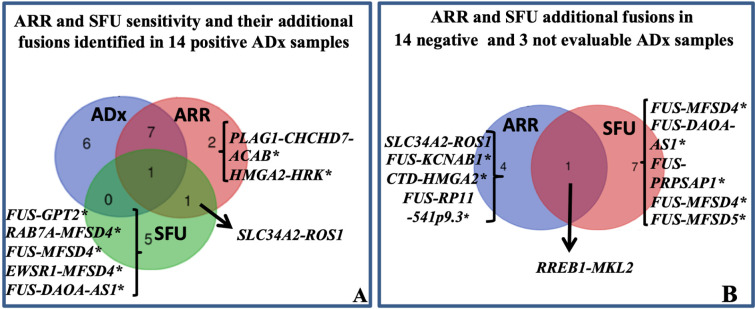
Sarcoma panel: ARR and SFU results. A: Venn diagram showing ARR and SFU sensitivity. The sensitivity was evaluated assuming that the 14 fusions identified by ADx were true positive. The fusion *SLC34A2-ROS1* was identified in sample 3 (Table 1) also harboring the EWSR1-TFCP2 translocation. B: Venn diagram showing the additional fusions identified by ARR and SFU in 14 negative and 3 not evaluable ADx cases. With the exception of *SLC34A2-ROS1* and ras responsive element binding protein 1 (*RREB1*)-*MKL2*, all the others translocations marked by * are unknown/unpublished. *CHCHD7*: coiled-coil-helix-coiled-coil-helix domain containing 7; *HRK*: harakiri, BCL2 interacting protein; *GPT2*: glutamic-pyruvic transaminase 2; *RAB7A*: RAB7A, member RAS oncogene family; *MFSD4*: major facilitator superfamily domain containing 4; *DAOA-AS1*: D-amino acid oxidase activator antisense RNA 1; *PRPSAP1*: phosphoribosyl pyrophosphate synthetase associated protein 1; *ACAB*: acetyl-CoA carboxylase beta; *KCNAB1*: potassium voltage-gated channel subfamily A regulatory beta subunit 1; *CTD*: Coats disease; *RP11*: retinitis pigmentosa 11

Differently from ADx, ARR and SFU allow to generate output independent of manufacturer pre-fixed quality controls. Conversely, they are not designed to detect exon skipping. Thus, all 31 samples could be successfully analyzed. Overall, 16 fusions in 16 of 31 (16/31: 52%) samples and 17 fusions in 15 (15/31: 48%) samples were detected by ARR and SFU, respectively (Table S2). Firstly, provided that all the 14 fusion transcripts identified by ADx were reliable as explained above, we expected to confirm such translocations among those identified by ARR and SFU. ARR confirmed 8/14 ADx fusions; out of the remaining 6 samples, ARR detected different fusions in 3 samples and no fusion in 3 cases. SFU confirmed one ADx fusion; of the remaining 13 samples, SFU detected different fusions in 8 cases and no fusions in 5 cases (Figure 2A, Table S2). Overall, assuming ADx as a reference method, ARR and SFU missed 6 and 13 translocations leading to a sensitivity of 57% (8/14) and 7% (1/14), respectively. To our knowledge, among the new fusions identified by ARR and SFU in the ADx-positive samples, only the solute carrier family 34 member 2 (*SLC34A2*)-*ROS1* (sample 3, Table 1, Table S2) is already described as a well-recognized partnership in carcinomas [18, 19], while all the others are unknown/unpublished or complex fusions involving more than 2 partners. Interestingly, the *SLC34A2*-*ROS1* is potentially actionable, but its presence in sample 3 was excluded by *ROS1* FISH.

#### ARR and SFU PPV and NPV in sarcoma panel

Among the 14 ADx-“no fusion” and the 3 ADx-“not evaluable” samples, 5 and 8 new fusions transcripts were identified by ARR and SFU respectively (Figure 2B, Table S2). Considering together all the additionally identified fusions by ARR and SFU, PPV of 61.5% and 7.1%, and NPV of 80% and 64%, respectively, can be obtained.

With the exception of *SLC34A2-ROS1* identified by ARR (sample 25, Table S2) and *RREB1-MKL2* (sample 29, Table S2), found by both ARR and SFU, all the others are unknown/unpublished. Since the *RREB1-MKL2* fusion was identified in one case for which residual FFPE material was available, we aimed at confirming its occurrence by FISH and RT-PCR. FISH confirmed *RREB1* translocation and RT-PCR confirmed its fusion with *MKL2* (data not shown). This chimera was already described in 20 cases of ectomesenchymal chondromyxoid tumor [20] and in one bi-phenotypic oropharyngeal sarcoma [21]. Overall, our findings provide evidence that either ARR and, moreover, SFU showed only partial accuracy, preventing their exclusive use in the routine diagnostic workflow. However, prompted by the evidence that the *RREB1-MKL2* translocation was properly called by both the algorithms in one case which was called as “not evaluable” by ADx, an additional assessment with such tools could be valuable in ADx-low quality samples, for the screening of potential rearrangements to be validated by FISH and/or RT-PCR.

### Lung panel

#### ADx showed loss of sensitivity in low quality samples

**Figure 3.**
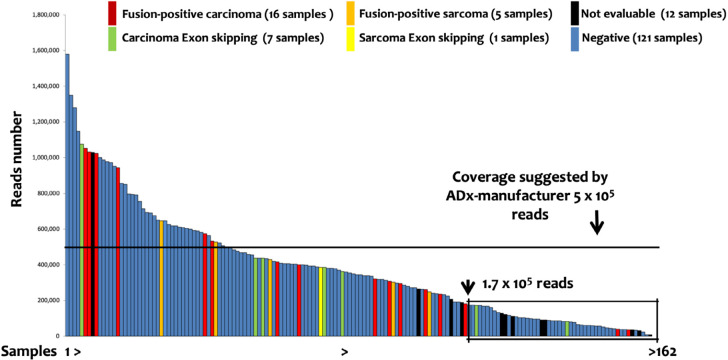
Lung Panel: ADx results. The 162 samples investigated by lung panel and analyzed by ADx are shown ordered on the basis of the number of the reads. The box evidenced the samples below 1.75 × 10^5^ reads, identified by a recursive contingency analysis (exact Fisher *t*-test) as a threshold below which the results cannot be considered reliable

One-hundred and fifty-nine patients (162 samples) were evaluated by the lung panel-ADx combination. The median reads number was 344,622, with a range from 7,854 to 1,578,000. Briefly: 29, 121, and 12 samples had positive (fusions or exons skipping detected), negative (no fusions), or not evaluable results, respectively. Among the 29 positive patients, 21 cases with fusion transcripts and 8 with exon-skipping in *NTRK3* (2), *NTRK2* (2), *MET* (3), and *RET* (1) genes were identified (Table 2). The detection of positive events in low reads-cases showed a decreasing trend in sensitivity (Figure 3) suggesting a possible increase of false negative results in samples with low coverage. Although ADx manufacturer’s instructions indicate 5 × 10^5^ reads as a threshold for reliable results, a recursive contingency analysis applied to our data suggested that this threshold might be decreased to 1.75 × 10^5^ reads. Indeed, below this reads number only 4 positive samples (2 translocations and 2 exon-skipping) were detected. One of the two translocations and one of the two exon-skipping were confirmed by FISH and direct sequencing respectively (samples 34 and 58, Table 2), while the other two could not be further investigated due to lack of further tissue (samples 46 and 56, Table 2).

**Table 2.** Table showing the gene fusions and exon skipping called by ADx in samples investigated by lung panel

**Samples**	Histology/Diagnosis*	**Lung panel ADx**	**FISH**
**32**	Aggressive glial neoplasia^a^	*KIF5C-ALK*	*ALK* confirmed
**33**	Poorly differentiated lung adenocarcinoma	*EML4-ALK*	*ALK* confirmed
**34**	Lung adenocarcinoma^a^	*KIF5B-RET*	*RET* confirmed^b^
**35**	Thyroid papillary carcinoma	*RET-NCOA4*	*RET* confirmed
**36**	Thyroid papillary carcinoma	*RELCH-RET*	*RET* confirmed
**37**	NTRK-rearranged spindle cell sarcoma	*TPM3-NTRK1*	*NTRK1* confirmed^b^
**38**	Liver adenocarcinoma^a^	*FGFR2-TACC3*	*FGFR2* non canonical
**39**	Secretory carcinoma of the parotid	*ETV6-NTRK3*	*ETV6*-*NTRK3* confirmed
**40**	Non small cell carcinoma with sarcomatoid features	*TPM3-NTRK1*	*NTRK1* not confirmed
**41**	Pilocytic astrocitoma^a^	*KIAA1549-BRAF*	Not done
**42**	Intracranic astrocitoma^a^	*KIAA1549-BRAF*	Not done
**43**	Lung adenocarcinoma	*EML4-ALK*	Not done
**44**	Thyroid papillary carcinoma^a^	*TPR-NTRK1*	Not done
**45**	Lung adenocarcinoma	*EML4-ALK*	Not done
**46**	Lung adenocarcinoma	*EML4-ALK*	Not done
**47**	NTRK rearranged inflammatory myofibroblastic tumor	*ETV6-NTRK3*	Not done
**48**	NTRK rearranged Spindle cell Sarcoma	*TPM3-NTRK1*	Not done
**49**	NTRK rearranged Spindle cell Sarcoma	*TPM3-NTRK1*	Not done
**50**	NTRK rearranged Spindle cell Sarcoma	*TPR-NTRK1*	Not done
**51**	Parotid carcinoma^a^	*ETV6-NTRK3*	Not done
**52**	NTRK rearranged spindle cell neoplasm	*TFG-NTRK3*	Not done
**53**	Talamic neoformation	NTRK2 exon5 skipping	Not achievable
**54**	Embryonal rhabdomyosarcoma	NTRK2 exon 5 skipping	Not achievable
**55**	Cerebellar HGNET-BCOR mutated^a^	NTRK3 exons 13–15 skipping	Not achievable
**56**	Frontal expansive lesion^a^	NTRK3 exon 16 skipping	Not achievable
**57**	Lung adenocarcinoma	MET exon 14 skipping	Not achievable
**58**	Lung adenocarcinoma	MET exon 14 skipping	Not achievable
**59**	Lung adenocarcinoma	MET exon 14 skipping	Not achievable
**60**	Neuroendocrine tumor (atypical carcinoid)	RET exons 4–7 skipping	Not achievable

a: external diagnosis. b: RNAseq was performed on surgical samples while FISH was done on CNBs prior surgery. *: According to WHO classification of Soft tissue and bone tumors [17] and to WHO classification of Thoracic tumors [22]; *KIF5C*: kinesin family member 5C; *EML4*: EMAP like 4; *RELCH*: RAB11 binding and LisH domain, coiled-coil and HEAT repeat containing; *TPM3*: tropomyosin 3; *TACC3*: transforming acidic coiled-coil containing protein 3; *TPR*: translocated promoter region, nuclear basket protein

#### ADx outcomes agreed with FISH results and/or patients’ diagnosis

FISH data were available in 9 out of the 21 fusions-positive samples and confirmed the translocation detected by ADx in all but one case (sample 40, Table 2), presenting with a *NTRK1* translocation. The gene fusions, identified by ADx in samples lacking FISH confirmation (sample 41–52, Table 2), were in line with the patient’s diagnosis: *KIAA1549-BRAF* in astrocytoma [23], *ALK* or *NTRK* translocations in thyroid papillary carcinoma [24], lung adenocarcinoma [25] as well as sarcomas NOS [26].

#### ROS1 and RET unbalanced translocations are not captured by NGS analysis

**Figure 4.**
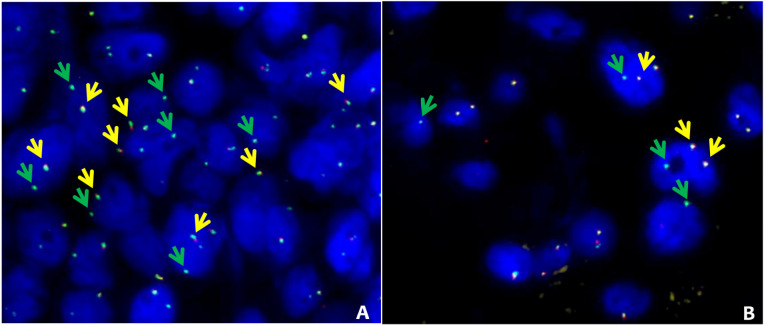
ROS1 and RET alternative FISH pattern. FISH results obtained in 2 patients with *ROS1* (sample 61, Table S3) and *RET* (sample 63, Table S3) with alternative FISH patterns. A: ROS1 break-apart fusion. The intact *ROS1* alleles are indicated by the yellow arrows (green plus red), while green arrows indicate isolated green signals corresponding to 3’ *ROS1* derivative. B: RET break-apart fusion. Intact *RET* alleles are indicated by the yellow arrows (green plus red), while green arrows indicate isolated 3’ RET derivatives. FISH break-apart probes SPEC ROS1 Dual Color Break Apart Probe Zyto*Light* (cat: Z-2144-50) and SPEC RET Dual Color Break Apart Probe Zyto*Light* (cat: Z-2148-200) were purchased from Zytovision (Williamsville, New York, USA) and were hybridized according to manufacturer’s instructions

FISH data, mainly related to *ROS1*, *ALK* and *RET* gene rearrangement status, were available in 31 of the 133 ADx negative or not evaluable samples (i.e. 121 negative and 12 not evaluable). In details, 28, 19, and 10 FISH were performed for *ROS1*, *ALK*, and *RET* respectively (Table S3). FISH break-apart strategy revealed the presence of an unbalanced pattern of rearrangement (recognized as translocation) [27, 28] characterized by the presence of intact *ROS1* (samples 61 and 62, Table S3) and *RET* (sample 63, Table S3) genes coupled with the presence of isolated 3’ *ROS1* and *RET* derivatives (Figure 4). Nonetheless, ROS1 IHC performed in sample 62 did not show any immunoreactivity (not supporting a *ROS1* productive translocation). Based on the available FISH (missing for RET) data, the ADx-*ROS1* translocations false negative detection rate can be estimated at 8% (2/28). Overall, our findings suggested that the lung panel-ADx pipeline is very reliable in clinical practice, albeit it may have limitations when dealing with samples yielding a non-optimal coverage (i.e. ≤ 1.75 × 10^5^ reads) or unbalanced translocations.

#### ARR but not SFU showed sensitivity close to ADx

**Figure 5.**
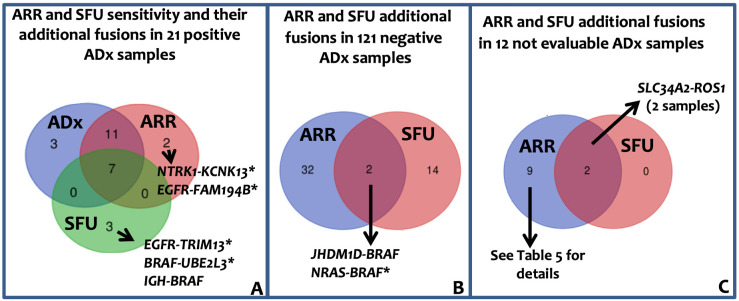
Lung panel: ARR and SFU results. A: Venn diagram showing ARR and SFU sensitivity. The sensitivity was evaluated assuming that the 21 fusions identified by ADx were true positive. Immunoglobulin heavy locus (*IGH*)-*BRAF* gene fusion was recently reported in one patient with Hairy Cells Leukemia BRAF-V600E negative [29]. B: Venn diagram showing the additional fusion identified by ARR band SFU in the 121 negative ADx samples. C: Venn diagram representing the fusion called by ARR and SFU in the 12 ADx not evaluable samples. *: fusions unknown/unpublished; *KCNK13*: potassium two pore domain channel subfamily K member 13; *TRIM13*: tripartite motif containing 13; *UBE2L3*: ubiquitin conjugating enzyme E2 L3; *JHDM1D*: jumonji C domain containing histone demethylase 1 homolog D; *NRAS*: NRAS proto-oncogene, GTPase; *FAM194B*: family with sequence similarity 194, member B

As for sarcoma panel, the lung panel raw data were analyzed through ARR and SFU algorithms. Since neither ARR nor SFU can detect exon-skipping, their sensitivity was evaluated in the 21 samples in which ADx pipeline has detected fusion transcripts. Overall, 18 fusions (86%) and 7 fusions (33%) in 21 samples were detected by ARR and SFU, respectively. ARR confirmed 18 fusions; out of the remaining 3 cases, ARR detected fusions different from ADx in one case and no fusion in 2 cases. SFU confirmed 7 fusions; out of the remaining 14 cases, SFU detected 3 chimeric transcripts different from ADx and no fusions in 11 cases (Figure 5A and Table S4 for details).

#### ARR and SFU PPV and NPV in lung panel

Among the 121 ADx-negative samples, 33 (in 30 patients) and 16 (in 16 patients) genes fusions were identified by ARR and SFU, respectively (Figure 5B, Table S5), leading to an ADx-ARR and ADx-SFU concordance in negative samples of 75% (90/121) and 87% (104/121) respectively. Overall, considering all the additionally identified fusions by ARR and SFU, PPV of 36.7% and 26.9%, and NPV of 99% and 89.5%, respectively, can be reported. Of the 50 fusions identified by ARR and SFU in ADx-negative patients, 3 were previously reported and 2 were detected by both the tools (Table S5). The complex fusions (composed of more than 2 genes) called by ARR and SFU in ADx-negative and not evaluable samples (Figure 5C, Tables S5 and S6) likely reflect the genomic complexity associated with carcinomas.

## Discussion

In this study, we report a cohort of solid tumor samples investigated for diagnostic purposes by RNAseq-based methodology in the context of the Institutional Molecular Tumor Board at the IRCCS Istituto Nazionale Tumori in Milan. The tumor samples have been analyzed by two different computational approaches, aimed at gene fusions detection for precision medicine. The first has been implemented using the ADx tool, provided by the manufacturer of the assay, and was run under stringent parameters to reduce false positive results. The second one was run to allow fusions detection also under “unfavorable” conditions (e.g., lower quality or lower number of reads), which are common in the case of FFPE samples. To this aim, two among the most commonly used tools for the investigation of chimeric transcript (ARR and SFU) have been chosen. Moreover, two different panels were used for cDNA libraries construction and have been analyzed herein: the FusionPlex^©^ sarcoma panel was applied in a number of STTs mainly for diagnostic purposes, while the FusionPlex^©^ lung panel was specifically used in clinical routine for the identification of molecular targets for precision medicine. To our knowledge, this represents the first comparison of different bioinformatics pipelines for the detection of fusion events for personalized treatment in a large real-world tumor cohort in the context of institutional Molecular Tumor Board. RNAseq data were furthermore compared with FISH, whenever available.

Overall, our results proved that the analysis performed by the manufacturer’s informatic pipeline on the sarcoma panel data was even more accurate and powerful than FISH (thanks to the possibility of capturing the partner loci of the tumor-associated genes), without any loss of specificity. On the other hand, the ADx pipeline on the lung panel highlighted that samples with low coverage are at higher risk of loss of sensitivity. For this reason, we deemed that the fundamental for the clinical practice to reinforce the output is obtained by a commercial pipeline (ADx) with those obtained by robust, publicly available, tools (such as ARR and SFU). It is nonetheless fundamental to underline that all the procedures described in the present study needed careful check by human resources, either by automated bioinformatics pipeline or by manual curation (i.e. visualizing outputs using tools like Integrated Genome Viewer in order to confirm the effectiveness of the identified fusion-associated reads).

To discuss our findings more in detail, all the fusions identified by ADx in sarcoma panel were in keeping with FISH data (when available), or IHC, or at least coherent with patient clinical features. Interestingly, three cryptic fusions (all missed by FISH) involving *SS18* and *CIC* were identified by ADx, suggesting an increased sensitivity of NGS. These results in particular strongly support the idea that the ADx constitutes a reliable tool for the detection of STT translocations in FFPE samples. Furthermore, our data suggest that the sensitivity was independent of sample quality, even whenever the quality metrics of the libraries felt just below the cut-off values according to the manufacturer’s criteria. This is in all likelihood granted by the intra-tumor molecular homogeneity [30] in the presence of high tumor cellularity in STT samples, which overall allows unraveling gene fusions even in more critical conditions. The ADx high sensitivity coupled with the independence from reads count supports the speculation that also the 14 negative STT samples were, actually, true negative. In line with this idea, all the performed FISH did not identify any rearrangement, supporting the high specificity of the pipeline. Interestingly, a large *ALK* deletion (Δ2-18) was evidenced by ADx in an *EWSR1-TFCP2* positive epithelioid rhabdomyosarcoma (in which IHC showed ALK overexpression and FISH the presence of an unbalanced *ALK* translocation characterized by the loss of the 5’ centromeric probe and the maintenance of 3’ telomeric probe. Similar *ALK* large deletions coupled with its over-expression were identified in a large series of TFCP2-translocated epithelioid rhabdomyosarcomas which, different from our case, did not show *ALK* gene rearrangement by FISH [31, 32]. Interestingly, unbalanced *ALK* translocation together with large deletion (Δ2-17) and ALK IHC over-expression was reported in a patient with systemic anaplastic lymphoma [33]. Combining our evidence with published data, we hypothesized that the observed ALK over-expression could be more likely due to genomic rearrangement at the *ALK* gene locus rather than gene fusion.

Previous reports, in the literature, indicated the occurrence of false negative results (e.g., 10% effective fusions were not identified in a set of 81 sarcoma samples analyzed by and ADx documented by Racanelli et al. [12]). For this reason, we tried to check the presence of chimeric transcripts, undetected by ADx, by applying two additional bioinformatic tools, ARR [9] and SFU [10]. To explain briefly the difference among the tools, ADx detects gene fusions by annotating the *de-novo* assembled RNA with basic local alignment search tool (BLAST) (ADx Analysis User Manual). This strategy is based on the assembly of short RNA sequences that are reciprocally contiguous in a full transcript (the “*de novo* assembly” step) and by their alignment (BLAST analysis) to the reference genome. Only the most abundant transcripts will be assembled [34]. To potentially increase the overall reliability, the data were re-analyzed with ARR and SFU. Both the tools are designed on STAR-seq aligners [35] and based on the “align-then-assemble” approach, which first aligns short RNA sequences reads to the genome (aware of the possible splicing events), and then reconstructs the transcripts. Differently from *de novo* assembly, this method can detect also less abundant transcripts [34]. Both ARR and SFU were reported to be among the most accurate (and fastest) methods for fusion detection on cancer transcriptome [11]. Surprisingly, among the 14 fusions identified by ADx, only 8/14 (57%) and 1/14 (7%) were confirmed by ARR and SFU respectively (only 1 fusion was shared between all methods); conversely, the major part of the new chimeric transcripts identified by ARR and SFU were unknown/unpublished. In contrast with previously reported data [11], our results showed a lower sensitivity of both methods in comparison with ADx. These findings prevent the use of these pipelines in diagnostic activity. However, in one sample not evaluable by ADx, despite characterized by high coverage, both ARR and SFU methods identified a fusion (*RREB1-MKL2*) that was missed by ADx and confirmed by FISH and RT-PCR. This result therefore suggests that, in peculiar cases, ARR and SFU can contribute to the detection of ADx-missed fusions, and prompts to further validations (FISH or PCR-based).

The FusionPlex^®^ lung panel has been applied for identifying a molecular target in a series of 162 FFPE samples obtained from 159 patients with solid cancers (mainly lung adenocarcinoma, but also various types of carcinoma). In this series, ADx identified 29 (18.2%) events, including 21 (13%) fusion transcripts and 8 (5%) exon skipping. In line with the manufacturers’ guidelines that suggest 5 × 10^5^ minimal reads count, a decreased sensitivity was observed in samples with low coverage. We are well aware of this read counts limitation: in clinical practice, however, this amount is not always achievable in FFPE samples from CNB or in cytology specimens that frequently present limited tumor area. Notably, in our series, a drop in ADx sensitivity was observed below 1.75 × 10^5^ reads. Below this threshold, the positive ADx outputs need to be confirmed by FISH and/or RT-PCR, while negative ones should be considered as not reliable. This conclusion is in line with data reported by Heydt and colleagues [36] on 18 fusion-positive cases, where ADx demonstrated high specificity with decreased sensitivity in samples with low-quality RNA. A similar conclusion was drawn in a series of FFPE *ROS1*-rearranged samples [37]. Indeed, not only pre-analytical and analytical conditions, but also the biology of carcinomas could contribute to increasing the percentage of false-negative, since it is well known that carcinoma, differently from STT [30], often express chimeric transcripts at low levels [1]. Other evidence is present in the literature that supports this, e.g., the comparison of RT-PCR with an NGS pipeline for the detection of *EML4-ALK* fusions in non-small cell lung cancer FFPE samples [38].

In addition to decreased sensitivity in samples with low coverage, ADx missed two *ROS1* and one *RET* un-balanced translocations. These cases warrant a separate discussion. All of them showed “atypical” *ROS1* and *RET* FISH patterns, with one fusion signal and one isolated 3’ signal without the corresponding 5’ signal. To our knowledge, to date, it is not yet clear if this pattern might be coupled with increased *ROS1* or *RET* expression (despite the *ROS1* IHC negative data). Although, according to *ROS1* and *RET* FISH guidelines, such a pattern should be considered positive [27, 28], those events can be due to deletions involving the 5’ portion of the genes locus but cannot be automatically associated with the presence of gene fusion. It is worth mentioning that also both ARR and SFU output the absence of the two *ROS1* and one *RET* translocations. The reason for this discrepancy between RNAseq, FISH, and IHC deserves further investigation.

Finally, as described for sarcoma panel, lung panel-ADx data were re-analyzed by ARR and SFU with higher overall inter-pipeline agreement than that observed in STT samples. In particular, ARR confirmed 18/21 (85%) ADx fusions and 90/120 ADx negative samples whereas, similarly to what was observed in STT, SFU confirmed only 7/21 (34%) translocations. In FusionPlex^©^ lung panel, therefore, ARR sensitivity was comparable to ADx one, leaving open the question of why ARR exhibited a better performance when applied with lung panels than sarcoma panel.

Despite the limited number of cases included in our cohort and the lack of a complete FISH validation, our data, obtained in a real-life series of FFPE samples, strongly support the use of NGS for the detection of fusion transcripts also in the presence of low-quality material. On the other hand, in samples that express chimeric genes at very low level (i.e. in lung carcinoma), we recommend to support the obtained results with other approaches, such as FISH and/or RT-PCR, provided that ADx, to date, represents a valuable and accurate pipeline for fusion detection and that the integration of more bioinformatic tools should be exploited to reinforce the workflow, particularly in the analysis of low-quality samples. Overall, our findings highlight the increasing need of structured bioinformatics support within public hospitals and standard diagnostic procedures.

## Abbreviations

ADx: ArcherDX^®^ Analysis suite
*ALK*: ALK receptor tyrosine kinase
ARR: Arriba
BAC: bacterial artificial chromosome
*BRAF*: B-Raf proto-oncogene
cat: catalog
*CIC*: capicua transcriptional repressor
CNBs: core needle biopsies
*EML4*: EMAP like 4
*ETV6*: ETS variant transcription factor 6
*EWSR1*: EWS RNA binding protein 1
FFPE: formalin-fixed paraffin-embedded
*FGFR1*: fibroblast growth factor receptor 1
FISH: fluorescence *in situ* hybridization
*FLI1*: friend leukemia virus integration 1
*FUS*: FUS RNA binding protein
IHC: immunohistochemistry
*MET*: MET proto-oncogene, receptor tyrosine kinase
*MKL2*: myocardin like 2
*NCOA2*: nuclear receptor coactivator 2
NGS: next-generation sequencing
NPV: negative predictive value
*NTRK3*: neurotrophic receptor tyrosine kinase 3
PPV: positive predictive value
*RET*: ret proto-oncogene
RNAseq: RNA-sequencing
*ROS1*: ROS proto-oncogene 1
*RREB1*: ras responsive element binding protein 1
RT-PCR: reverse-transcription polymerase chain reaction
SFU: spliced transcripts alignment to reference fusion
*SLC34A2*: solute carrier family 34 member 2
*SS18*: SS18 subunit of BAF chromatin remodeling complex
STAR: spliced transcripts alignment to reference
*STAT6*: signal transducer and activator of transcription 6
STTs: soft tissue tumors
*TFCP2*: transcription factor CP2
*YWHAE*: tyrosine 3-monooxygenase/tryptophan 5-monooxygenase activation protein epsilon

## Supplementary materials

The supplementary materials for this article are available at: https://www.explorationpub.com/uploads/Article/file/1002102_sup_1.pdf.

## Declarations

### Availability of data and materials

The raw data supporting the conclusions of this manuscript will be made available by the authors (luca.agnelli@istitutotumori.mi.it), without undue reservation, to any qualified researcher.

### Copyright

© The Author(s) 2022.
