# Supplementary material for: Targeted RNA-sequencing analysis for fusion transcripts detection in tumor diagnostics: assessment of bioinformatic tools reliability in FFPE samples
Source: Explor Target Antitumor Ther. 2022 Oct 27;3(5):582–97. doi: 10.37349/etat.2022.00102 (PMC9630092; doi:10.37349/etat.2022.00102)
Supplement: Supplementary file 1 [file etat-03-1002102-s001.pdf]

Supplementary Figure 1. ALK unbalanced translocation

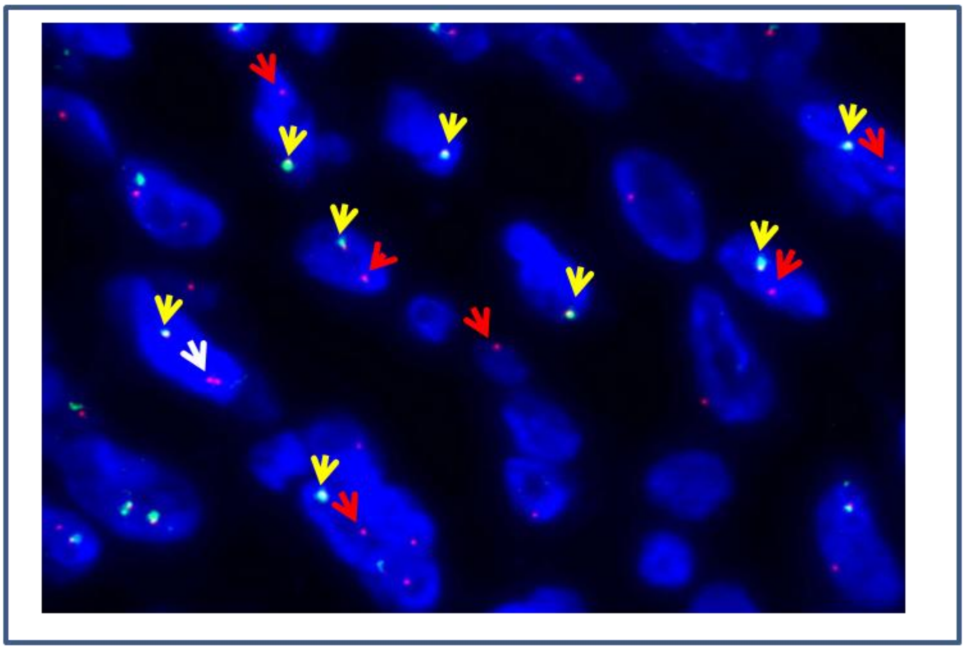

The ALK unbalanced translocation in Epithelioid Rhabdomyosarcoma (sample n3, table 1) was demonstrated by FISH ALK break-apart fusion. The intact ALK alleles are indicate by the yellow arrows (green plus red signals) while red arrows indicate isolated red signals corresponding to 3'ALK derivatives. ALK Break Apart Probe was purchased from Vysis (cat: o8N16-001, Abbot Park, Illinois USA) and were hybridized according to manufacturer instructions

Supplementary Table 1. Table showing the probes used for FISH experiments

Gene	Company	Probe Name
ALK	Abbott Molecular	Vysis ALK Dual Color, Break Apart FISH Probe
SS18	Abbott Molecular	Vysis SS18 Dual Color, Break Apart Probe
EWS	Abbott Molecular	Vysis EWSR1 Dual Color, Break Apart Probe
ROS1	Zytovision	ZytoLight SPEC ROS1 Dual Color, Break Apart Probe
EWSR1-FLI1	Zytovision	ZytoLight PEC EWSR1/FLI1 TriChec Probe
FGFR2	Zytovision	ZytoLight SPEC FGFR2 Dual Color, Break Apart Probe
FUS	Zytovision	ZytoLight SPEC FUS Dual Color, Break Apart Probe
RET	Zytovision	ZytoLight SPEC RET Dual Color, Break Apart Probe
SS18 SSX1	Zytovision	ZytoLight SPEC SS18/SSX1 TriCheck Probe
TFE3	Zytovision	ZytoLight SPEC TFE3 Dual Color, Break Apart Probe
YWHAE	Zytovision	ZytoLight SPEC YWHAE Dual Color, Break Apart Probe

ATF1	BAC. In house labelled	RP11-834H3, RP11-787D7, RP11-991C19
BCOR	BAC. In house labelled	RP11-429N5, RP11-320G24
CIC	BAC. In house labelled	RP11-317E13, RP11-374A11
CREB1	BAC. In house labelled	RP11-703O8, RP11-237A5
ETV6	BAC. In house labelled	RP11-75N23, RP11-418C2
NTRK3	BAC. In house labelled	RP11-846G5, RP11-285I14, RP11-121M4
NR4A3	BAC. In house labelled	RP11-30L7, RP11-30N20
NTRK1	BAC. In house labelled	RP11-180B22, RP11-1038N13
NCOA2	BAC. In house labelled	RP11-183D21, RP11-774K7, RP11-479K21
PHF1	BAC. In house labelled	RP11-602P21, RP11-175A4.
WWTR1	BAC. In house labelled	RP11-941L15, RP11-1151O19

BA: break apart, F: fusion

Supplementary Table 2. Table showing the comparison between the fusions called by ADx, ARR and SFU in samples investigated by sarcoma panel

Samples	Sarcoma panel ADx	Sarcoma panel ARR	Sarcoma panel SFU	FISH
**1**	*ETV6-NTRK3*	*PLAG1-CHCHD7–ACACB**	*FUS-GPT2**	*ETV6-NTRK3* confirmed
**2**	*EWSR1- PATZ1*	*EWSR1- PATZ1*	*RAB7A-MFSD4**	*EWSR1* translocated
**3**	*EWSR1-TFCP2*	*SLC34A2-ROS1*	*SLC34A2-ROS1*	*EWSR1* translocated
	ALK Δ*2-18*			*ALK* unbalanced translocation
**4**	*EWSR1-NR4A3*	no fusions	no fusions	*EWSR1 -NR4A3* confirmed
**5**	*EWSR1-FLI1*	no fusions	*FUS-MFSD4**	*EWSR1-FLI1* confirmed
**6**	*EWSR1-CREM*	*EWSR1-CREM*	no fusions	*EWSR1* translocated
**7**	*YWHAE-NUT2*	*HMGA2-HRK**	no fusions	*YWHAE* translocated
**8**	*SS18 -SSX4*	*SS18 -SSX4*	*EWSR1-MFSD4**	*SS18* intragenic rearrangement
**9**	*SS18-SSX1*	*SS18-SSX1*	*FUS-MFSD4**	*SS18* not translocated
**10**	*CIC-DUX4*	*CIC-DUX4*	*FUS-MFSD4**	CIC not translocated
**11**	*EWSR1-CREB3L2*	*EWSR1-CREB3L2*	no fusions	Not done
**12**	*EWSR1-FLI1*	*EWSR1-FLI1*	*FUS-DAOA-AS1**	Not done
**13**	*NAB2-STAT 6*	no fusions	no fusions	Not detectable by FISH
**14**	*ETV6-NTRK3*	*ETV6-NTRK3*	*ETV6-NTRK3*	Not done
**15**	no fusions	no fusions	*FUS-MFSD4**<br>*FUS-DAOA-AS1**<br>*FUS-PRPSAP1**	*SS18, EWSR1, FUS* negative
**16**	no fusions	no fusions	no fusions	*WWTR1* negative[a]
**17**	no fusions	no fusions	no fusions	*SS18, BCOR, CIC* negative
**18**	no fusions	no fusions	no fusions	*EWSR1, FUS* negative[a]
**19**	no fusions	no fusions	*FUS-PRPSAP1**	*SS18* negative
**20**	no fusions	no fusions	*FUS-PRPSAP1**	*EWSR1, FUS, ATF1, CREB1* negative

**21**	no fusions	no fusions	no fusions	Not done
**22**	no fusions	no fusions	no fusions	*EWSR1, BCOR, FUS, CIC, NCOA2* negative[a]
**23**	no fusions	no fusions	no fusions	Not done
**24**	no fusions	no fusions	no fusions	Not done
**25**	no fusions	*SLC34A2-ROS1*	*FUS-MFSD4**	FUS negative[a]
**26**	no fusions	no fusions	no fusions	Not done
**27**	no fusions	no fusions	no fusions	ALK negative
**28**	no fusions	*FUS-KCNAB1**	*FUS-MFSD5**	*SS18, EWSR1, BCOR, CIC, TFE3* negative
**29**	not evaluable	*RREB1-MKL2*[b]	*RREB1-MKL2*[b]	*PHF1,FUS,CIC,BCOR,EWSR1,NR4A3* negative
**30**	not evaluable	*CTD-HMGA2**	no fusions	*EWSR1, NCOA2, CIC, SS18, BCOR* negative
**31**	not evaluable	*FUS-RP11-541P9.3**	no fusions	Not done
**Total**	**14 fusions**	**16 fusions**	**17 fusions**	

a: The FISH were performed on different samples respect to those used for NGS. b: The fusion was confirmed by FISH and RT-PCR that were performed after the *RREB1-MKL2* fusion identification by ARR and SFU.* Unknown/unpublished

Supplementary Table 3: table showing the ROS1, ALK and RET FISH performed in 31 out of 121 ADx negative samples

**Samples**	**FISH**		
	ROS1	***ALK***	***RET***
**61**	**Translocated**	neg	neg
**62**	**Translocated**	neg	neg
**63**	neg	neg	**Translocated**
**64**	neg	Not done	neg
**65**	neg	Not done	Not done
**66**	neg	neg	Not done
**67**	neg	Not done	Not done
**68**	neg	Not done	Not done
**69**	neg	neg	Not done
**70**	neg	neg	Not done
**71**	neg	neg	neg
**72**	neg	neg	neg
**73**	neg	neg	Not done
**74**	Not done	neg	Not done
**75**	Not done	Not done	neg
**76**	Not done	neg	Not done
**77**	neg	Not done	neg
**78**	neg	Not done	Not done
**79**	neg	neg	Not done
**80**	neg	neg	Not done

**81**	neg	neg	Not done
**82**	neg	Not done	Not done
**83**	neg	neg	Not done
**84**	neg	neg	Not done
**85**	neg	Not done	Not done
**86**	neg	Not done	neg
**87**	neg	neg	Not done
**88**	neg	Not done	neg
**89**	neg	Not done	Not done
**90**	hem del	neg	Not done
**91**	neg	neg	Not done
**total samples:31**	**total *ROS1*: 28**	**total *ALK*: 19**	**total *RET*: 10**

All the translocation detected by FISH were unbalanced. neg: Negative; hem del: ROS1 hemizygote deletion.

Supplementary Table 4. Table showing the fusion genes identified by ARR and SFU in the 21 ADx positive samples

Samples	Lung panel ADx	Lung panel ARR	Lung Panel SFU	FISH
32	*KIF5C-ALK*	*KIF5C-ALK*	no fusions	*ALK* confirmed
33	*EML4-ALK*	*EML4-ALK*	no fusions	*ALK* confirmed
34	*KIF5B-RET*	*KIF5B-RET*	no fusions	*RET* confirmed
35	*RET-NCOA4*	*RET-NCOA4*	no fusions	*RET* confirmed
36	*KIAA1468-RET*	*NTRK1- KCNK13** *EGFR-FAM194B**	no fusions	*RET* confirmed
37	*TPM3-NTRK1*	*TPM3-NTRK1*	TPM3-NTRK1	*NTRK1* confirmed
38	*FGFR2-TACC3*	*FGFR2-TACC3*	*FGFR2-TACC3*	*FGFR2* confirmed
39	*ETV6-NTRK3*	no fusions	no fusions	*ETV6-NTRK3* confirmed
40	*TPM3 -NTRK1*	*TPM3 -NRTK1*	*EGFR-TRIM13**	*NTRK1* not confirmed
41	*KIAA1549-BRAF*	*KIAA1549-BRAF*	*BRAF-UBE2L3**	Nd
42	*KIAA1549-BRAF*	*KIAA1549-BRAF*	no fusions	Nd
43	*EML4-ALK*	*EML4-ALK*	*EML4-ALK*	Nd
44	*TPR-NTRK1*	*TPR-NTRK1*	no fusions	Nd
45	*EML4-ALK*	*EML4-ALK*	no fusions	Nd
46	*EML4-ALK*	no fusions	no fusions	Nd
47	*ETV6-NTRK3*	*ETV6-NTRK3*	*ETV6-NTRK3*	Nd
48	*TPM3-NTRK1*	*TPM3-NTRK1*	*TPM3-NTRK1*	Nd
49	*TPM3-NTRK1*	*TPM3-NTRK1*	*TPM3-NTRK1*	Nd
50	*TPR-NTRK1*	*TPR-NTRK1*	no fusions	Nd
51	*ETV6-NTRK3*	*ETV6-NTRK3*	*ETV6-NTRK3*	Nd
52	*TFG-NTRK3*	*TFG-NTRK3*	*IGH-BRAF*[a]	Nd

a: _IGH-BRAF_ gene fusion was recently reported in one patient with Hairy Cells Leukemia V600E negative (_Thompson et al, Leukemia e Lymphoma 2020; 61:8, 2024-2026_). *: unknown-unpublished fusions; Nd: not done

Supplementary Table 5. Table showing the gene fusion called by ARR and SFU in ADx negative samples

Samples	Lung panel ADx	Lung panel ARR	Lung panel SFU
61 (supp. Tab2)	no fusions	_EGFR-NTRK3*_	no fusions
64 (supp. Tab2)	no fusions	_BRAF-KIAA_[a]	_BRAF-RASA1*_
65 (supp. Tab2)	no fusions	_TNS3-ROS1*_	no fusions
66 (supp. Tab2)	no fusions	_SMARCB1-PDGFRA*_	_FGFR3-MFSD4*_
69 (supp. Tab2)	no fusions	_CHMP2A->CTD-UQCRFS1*_	no fusions
		SOX6->BRAF*	
70 (supp. Tab2)	no fusions	_RP11-MYOZ2->ROS1*_	no fusions
71 (supp. Tab2)	no fusions	_PRMT3-EGFR*_	no fusions
92	no fusions	_JHDM1D-BRAF_[b]	_JHDM1D-BRAF_
93	no fusions	_NRAS-BRAF*_	_NRAS-BRAF*_
94	no fusions	_SLC34A2-ROS1_[c]	no fusions
95	no fusions	_KDR - RET*_	no fusions
96	no fusions	_MET-FGFR2*_	no fusions
97	no fusions	_ATM-FGFR2*_	no fusions
98	no fusions	_FLT3 -SMAD4*_	no fusions
99	no fusions	_EGFR -MFN2*_	no fusions
100	no fusions	_MET-SEMA3A*_	no fusions
101	no fusions	_RP11- SST-BRAF*_	no fusions
102	no fusions	_CLTCL1-ALK*_	no fusions
103	no fusions	_TNS3 - ROS1*_	no fusions
104	no fusions	_RP11-MYOZ2->ROS1*_	_BAI2-EGFR*_
105	no fusions	_ZNF609 - KRAS*_	no fusions
106	no fusions	_RP11-MYOZ2->ROS1*_	no fusions
107	no fusions	_RP11-MYOZ2->ROS1*_	no fusions
108	no fusions	_TRIO - FGFR1*_	no fusions
109	no fusions	_DSCC1-MET*_	no fusions
110	no fusions	_RP11-FGFR2*_	no fusions
		MET->CTNND1- OR5BA1P*	
111	no fusions	_ETV6-KRAS*_	no fusions
112	no fusions	_RP11-MYOZ2->ROS1*_	no fusions
113	no fusions	_RP11- BRAF*_	no fusions
		FGFR2 - GABRG1*	

114	no fusions	*HPSE2-ROS1**	no fusions
116	no fusions	no fusions	*RAB7A-MFSD4**
117	no fusions	no fusions	*RAB7A-MFSD4**
118	no fusions	no fusions	*RAB7A-MFSD4**
119	no fusions	no fusions	*MET-MFSD4**
120	no fusions	no fusions	*RAB7A-MFSD4**
121	no fusions	no fusions	*RAB7A-MFSD4**
122	no fusions	no fusions	*CCNC-KRAS**
123	no fusions	no fusions	*FUS-B3GALT5**
124	no fusions	no fusions	*MBD1-MET**
122	no fusions	no fusions	*RAB7A-ZNF618**
125	no fusions	no fusions	*RORA-BRAF**

a: *BRAF-KIAA* (sample 64) was identified in pilocytic astrocitoma (*Balasubramanian et al J Clin Neurosci. 2020 ;79:269-271*). b: *JHDM1D-BRAF* (sample 92) was previously reported in gastric cancer [*Sase et al Mol Cancer Ther. 2018;17(10):2217-2225*]. c: while *SLC34A2-ROS1* (case 94) has been reported in carcinomas [*Lee et al, Mod Pathol. 2015;(4):468-79 and Davies et al. Clin Cancer Res. 2012;18(17):4570-9*]. The prevalence of *RAB7A* and *MFSD4* genes in the fusions identified by SFU, strongly suggest that were false positive results. Supp. Tab2: sample already reported in Supplementary Table 3. * All these fusions were previously unreported/unpublished

Supplementary Table 6. Gene fusion identified by ARR and SFU in the 12 ADx not evaluable samples

Samples	Lung panel ADx	Lung panel ARR	Lung panel SFU
N63 (supp tab2)	not evaluable	*SLC34A2-ROS1*	*SLC34A2-ROS1*
N73 (supp.tab2)	not evaluable	*BRAF-RDX**<br>*ABHD11-CLDN3->NTRK2**<br>*MET->CTD-TMEM86B**<br>*AL356154.1-NRG3->NTRK3**<br>*PLSCR4-RP11->EGFR**	no fusions
N88 (supp.tab2)	not evaluable	no fusions	no fusions
N89 (supp.tab2)	not evaluable	no fusions	no fusions
126	not evaluable	*SLC34A2-ROS1*	*SLC34A2-ROS1*
127	not evaluable	*PSMD6,RP11-129B22.1-NRG1**	no fusions
128	not evaluable	*NRG1->CSNK1A1L-RN7SKP1**<br>*NTRK2->RP11- RP11**	no fusions

129	not evaluable	*RET-KIF5B*[a]	no fusions
130	not evaluable	no fusions	no fusions
131	not evaluable	no fusions	no fusions
132	not evaluable	no fusions	no fusions
133	not evaluable	no fusions	no fusions

a: The *RET-KIF5B* translocation (case n 129) was previously published (*Velcheti V et al, Thorac Oncol. 2017;12(7):e98-e99.v*) in a lung adenocarcinoma patient. Supp. Tab2: sample already reported in Supplementary Table 3. *: all these fusions were previously unreported/unpublished

The parameters used for Arriba:

```
export version=1.8.1
export sample=$PATIENT.fastq
export folder=$PATIENT
STAR    --runThreadN 8
        --genomeDir
        $HOME/genome/GRCh37_gencode_v19_CTAT_lib_Apr032020.plug-n-play/ctat_ge
        nome_lib_build_dir/ref_genome.fa.star.idx
        --genomeLoad NoSharedMemory
        --readFilesIn $HOME/fastq/$sample
        --outTmpDir $HOME/NGSeqINT/temp$folder
        --outStd BAM_Unsorted
        --outSAMtype BAM Unsorted
        --outSAMunmapped Within
        --outBAMcompression 0
        --outFilterMultimapNmax 1
        --outFilterMismatchNmax 3
        --chimSegmentMin 10
        --chimOutType WithinBAM SoftClip
        --chimJunctionOverhangMin 10
        --chimScoreMin 1
        --chimScoreDropMax 30
        --chimScoreJunctionNonGTAG 0
        --chimScoreSeparation 1
        --alignSJstitchMismatchNmax 5 -1 5 5
        --chimSegmentReadGapMax 3 |
arriba   -x /dev/stdin
        -o $HOME/analysis/$folder/ARRIBA.fusions.tsv
        -O $HOME/analysis/$folder/ARRIBA.fusions.discarded.tsv
```

-a
$HOME/genome/GRCh37_gencode_v19_CTAT_lib_Apr032020.plug-n-play/ctat_genome_lib_build_dir/ref_genome.fa
-g
$HOME/genome/GRCh37_gencode_v19_CTAT_lib_Apr032020.plug-n-play/ctat_genome_lib_build_dir/ref_annot.gtf
-b
$HOME/arriba/arriba_v2.0.0/database/blacklist_hg19_hs37d5_GRCh37_v2.0.0.tsv.gz
